# Atteintes osseuses caricaturales d'un myélome multiple chez une octogénaire

**DOI:** 10.11604/pamj.2015.22.148.8098

**Published:** 2015-10-16

**Authors:** Ines Kéchaou, Imène Boukhris

**Affiliations:** 1 Service de Médecine Interne B, Hôpital Charles Nicolle, Tunis, Tunisie

**Keywords:** Myélome multiple, géode, atteinte radiologique, multiple myeloma, geode, radiological involvement

## Image en medicine

Une patiente âgée de 87 ans, hypertendue depuis 30 ans, était admise pou bilan étiologique de douleurs osseuses au niveau du bassin, résistantes aux antalgiques usuels évoluant depuis 4 mois avant son admission dans un contexte d'altération de l’état général. Au bilan biologique, elle avait une anémie normochrome normocytaire (Hb: 8,5 g/dL, VGM: 86,1 µ3), une créatinine à 103 µmol/l avec une clairance à 22 ml/mn, une VS à 93 mm, une hyper gammaglobulinémie d'allure monoclonale à 31,45 g/L. la calcémie était normale à 2,5 mmol/L. Le bilan radiologique des os douloureux a révélé de multiples géodes à l'emporte pièce au niveau du bassin surtout au niveau des ailes ischio-pubiennes et du tiers supérieur du fémur et une fracture tassement au niveau de la 12ème vertèbre thoracique. La ponction sternale a montré une infiltration médullaire estimée à au moins 40% par une population plasmocytaire franchement dystrophique. L'immunoélectrophorèse des protéines sérique a objectivé la présence d'une immunoglobuline monoclonale de type IgG Kappa. La recherche de protéinurie de Bence-Jones était négative. Le diagnostic de myélome multiple stade III A a été alors retenu. Vu l'importante atteinte osseuse, une prévention des chutes s'impose chez cette patiente à fin de réduire le risque de fractures.

**Figure 1:**
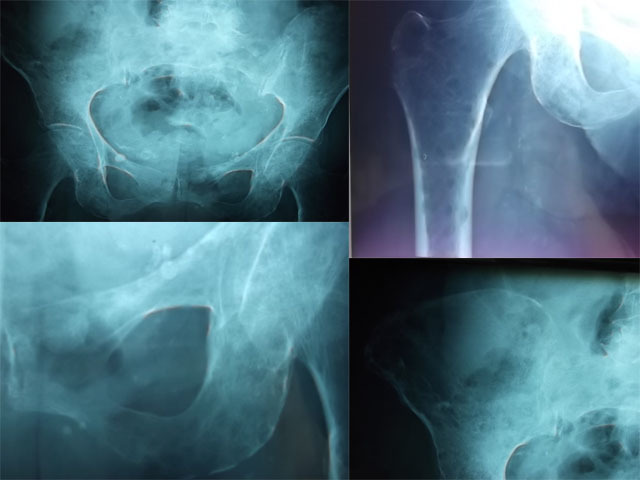
Multiples géodes au niveau du bassin et du tiers supérieur du fémur

